# Fusion proteins of biologic agents in the treatment of rheumatoid arthritis (RA): A network meta-analysis

**DOI:** 10.1097/MD.0000000000026350

**Published:** 2021-06-18

**Authors:** Mingcai Wu, Mengjun Tao, Quanhai Wang, Xiaohua Lu, Hui Yuan

**Affiliations:** a Department of Biochemistry and Molecular Biology; b Department of Epidemiology and Biostatistics; c Functional experiment and training center, School of Public Health, Wannan Medical College, Wuhu, China.

**Keywords:** abatacept, anakinra, biologic agents, etanercept, meta-analysis, rheumatoid arthritis

## Abstract

**Background:**

To evaluate the efficacy of fusion proteins biologics (Etanercept (ETN), Anakinra (ANA), and Abatacept) combinations in the treatment of rheumatoid arthritis (RA) using network meta-analysis to rank those according to their performance medicines. The performance of these processes is ranked according to the results of the analysis and an explanatory study of the possible results is carried out.

**Methods:**

Multiple databases including PubMed, EMBASE, and Cochrane Library were used to identify applicable articles and collect relevant data to analyze using STATA (14.0) software. The literature included in this study was divided into a combination of a placebo, methotrexate (MTX), and an observation group (1 of the 3 drugs). The last search date was December 12, 2019.

**Results:**

A total of 19 eligible randomized controlled trials of fusion proteins biologics were identified, a total of 1109 papers were included, and the results showed that the ETN + MTX had the highest probability of being the most clinically efficacious intervention, with a surface under the cumulative ranking curve of 91.6, was significantly superior (*P* < .05). Patients who had received ETN or ETN + MTX or ANA had effective compared with patients who had received placebo (95% CI 1.28%–8.47%; 1.92%–19.18%; 1.06%–10.45%).

**Conclusions:**

1. The combination of ETN and MTX had the highest probability of optimal treatment compared to other drugs and 2. ENT, ENT + MTX, and ANA were effective in the treatment of RA compared to placebo.

## 1 Introduction

Rheumatoid arthritis (RA) is a dynamic process with systemic autoimmune disease characterized by synovial inflammation, which is influenced by factors such as genetic, environmental, and hormonal factors.^[1]^ Recent studies have known that the key factor in destroying RA is the synovial fibroblasts in the hyperplastic synovial intima.^[2]^ A series of events, including pain and joint destruction, are caused by the progressive inflammation of synovial joints.^[3,4]^ The physical function and quality of life in RA patients are seriously threatened by joint destruction.^[5]^ This disease frequently occurs among women than men, which affects about 1% of the population with 25 to 50 new cases occur annually in a population of 100,000.^[6,7]^ Almost all RA patients need to be treated to delay or stop the development of the disease, or/and control the disease performance and reduce the disease burden.^[8]^ RA treated by biological agents has the characteristics of better symptom control and disease alleviation, compared with traditional medicines such as methotrexate (MTX).^[9]^ The currently available biological agents for RA can be classified as fusion proteins and monoclonal antibodies.^[10]^ The fusion proteins are composed of a receptor portion of the target molecule and the Fc region of the immunoglobulin.^[11]^ The currently available fusion proteins include Etanercept (ETN), Anakinra (ANA), and Abatacept (ABA). A series of studies have shown that single-agent and combination medicines have an efficient effect on RA, but the best optimal treatment for the fusion protein of RA is still unknown. Therefore, it is necessary to explore the biological effects of fusion protein drugs for the treatment of RA, and the effect of different fusion protein combinations in the treatment of RA, for providing a theoretical basis for the treatment of RA with biological therapy. It is necessary to explore the effects of different fusion protein combinations on the treatment of RA, for providing a theoretical basis for the biological treatment of RA.

Network meta-analysis (NMA) is an upgraded version of the traditional meta-analysis.^[12]^ It can comprehensively evaluate and classify multiple interventions simultaneously, especially in comparison with the effects of indirect different interventions using indirect controls. The aim of this study was to evaluate the efficacy of fusion proteins combinations in the treatment of RA using NMA to rank those according to their performance medicines (placebo, MTX, ETN, placebo + ETN, placebo + ANA, ANA + MTX, ABA + MTX, and ETN + MTX). It can identify the best way to treat RA with fusion proteins and help us understand the effective mechanism of disease treatment.

## 2 Methods

### 2.1 Search strategy

The databases used for this study included Pubmed, EMBASE, and Cochrane Library, which were searched before December 12, 2019, use Etanercept or Anakinra or Abatacept and rheumatoid arthritis with titles. By screening the titles and abstracts to ascertain whether the studies were met predefined selection criteria. Two reviewers screened the title and abstract of the paper as well as the potential related full-text articles. Then the data were extracted from the selected studies, including study characteristics, patient characteristics, and outcomes.

### 2.2 Inclusion and exclusion criteria

The inclusion criteria were as follow: (a) randomized controlled trials (RCTs); (b) all treatments or combinations can only be placebo or MTX or fusion proteins; (c) all patients with RA who met American College of Rheumatology (ACR) criteria for RA.^[13]^; (d) efficacy evaluation indicators were assessed using ACR20% (ACR20); (e) the language is English; and (f) multiple time nodes are taken only once for nearly 1 year.

The exclusion criteria were as follows: (a) biosimilar in RA; (b) other medications or combinations e) cost-effectiveness and cost-benefit evaluations; (c) case reports, systematic reviews; (d) animal experiments, cross-experimental studies; and (e) comparison before and after drug treatment, or no data available for analysis.

### 2.3 Efficacy evaluation criteria

Outcome indicators were included ACR 20 that defined as an improvement of 20% or more from baseline, in 3 of the following 5 domains: (a) a comprehensive assessment of arthritis activity by the patient; (b) a comprehensive assessment of arthritis activity by doctors; (c) patient assessment of arthritis pain. The assessment also included the number of joints (analysis from 66 joints) and the joint swelling (analysis from 68 joints); (d) health assessment questionnaire score; and (e) inflammatory markers, such as erythrocyte sedimentation rate or C-reactive protein.^[14]^ An ACR20% is defined as a 20% improvement in tender and swollen joint counts and the same level of improvement in 3 of the 5 domains.

### 2.4 Data extraction and quality evaluation

Literature and extraction were performed independently by 2 reviewers, based on the inclusion and exclusion criteria, including the following: (a) characteristics of the publication; (b) data quality of the publication; and (c) result indicator selection. The quality of the publications was evaluated using the Jadad quality scoring standard. Jadad scoring scale was consists of 3 items pertaining to descriptions of randomization, masking, and dropouts, and withdrawals in the report of an RCT. The scale ranges from 0 to 5, with higher scores indicating better reporting. High-quality trials scored more than 2 out of a maximum possible score of 5.^[15]^

### 2.5 Statistical analysis

Using commands of the network package in stata (14.0), the network, evidence contribution, predictive interval (*PrI*), funnel, and ranking plots were constructed. The efficacy of the intervention was ranked based on the surface values under the cumulative ranking (SUCRA) curve. The selected indicator was the count data, and *OR* is used as the combined effect, with a confidence interval (CI) set to 95%. A value of *P* < .05 was considered to be statistically significant.

### 2.6 Ethical statement

As this meta-analysis was based on previously published studies ethical approval was not necessary.

## 3 Results

### 3.1 Characteristics of included studies

**Figure 1:**
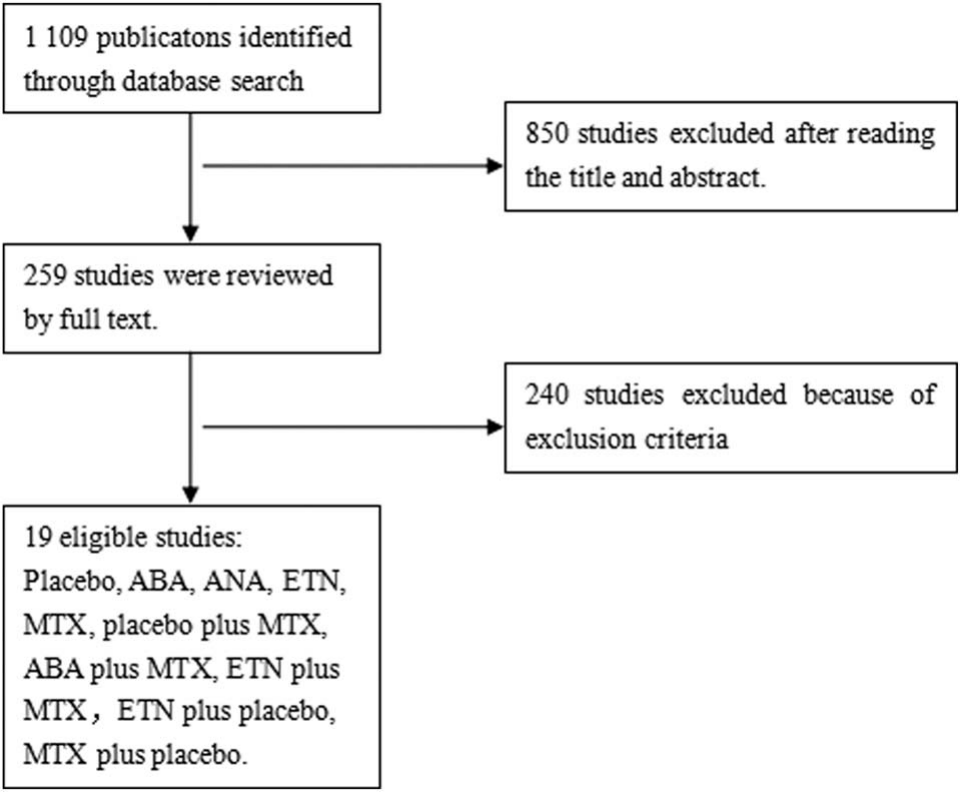
Flow diagram of the selected details of included publications.

Initially, 1109 potentially relevant articles were screened using relevant words. Among them 850 were excluded. After the full-text review of the studies, 240 studies were excluded due to exclusion criteria. Finally, 19 eligible studies are included. Figure 1 shows the selection process of published trials for this systematic review. A total of 19 RCTs involving 6812 patients were ultimately included in this analysis (placebo, ANA, ABA, ETN, MTX, placebo + MTX, ANA + MTX, ETN + MTX, ETN + placebo, MTX + placebo). All studies meet the inclusion criteria of the literature and show the basic characteristics of the studies are summarized in Table 1.

**Table 1:** Basic information of included studies in the network meta-analysis.

Study	Country	Cases	Control	Cases	Total cases	Control	Total Control	Duration of follow-up (mouth)	Jadad quality score
Nuki et al (2002)^[16]^	Europe	C	A	84	233	11	76	6	4
Cohen (2004)^[17]^	USA	F	J	95	250	55	251	6	3
Cohen et al (2002)^[18]^	Canada, Australia, USA	F	J	68	269	7	48	6	4
Emery et al (2015)^[19]^	USA, Europe	D	J	50	116	53	116	12	4
		G		73	119				
Westhovens et al (2014)^[20]^	Belgium	G	J	100	152	19	67	12	4
Takeuchi et al (2013)^[21]^	Japan	G	J	89	128	14	66	6	4
Matsubara et al (2018)^[22]^	Japan	G	J	153	203	56	202	4	4
Schiff et al (2008)^[23]^	USA	G	J	104	156	46	110	6	4
Kremer et al (2006)^[24]^	USA	G	J	294	433	87	219	12	4
Kremer et al (2005)^[25]^	USA	G	J	72	115	43	119	12	4
Weinblatt et al (2007)^[26]^	USA	G	I	41	85	11	36	12	4
van Riel et al (2006)^[27]^	Denmark, Finland, France, etc	E	H	110	155	102	152	4	3
Kameda et al (2010)^[28]^	Japan	E	H	44	69	66	73	6	4
Keystone et al (2004)^[29]^	USA, Canada	E	A	182	367	10	53	2	4
Bassiouni et al (2018)^[30]^	African, Middle Eastern	E	A	18	22	20	29	6	3
van der Heijde et al (2006)^[31]^	The Netherlands	E	B	167	223	162	228	24	4
		H		199	231				
Takeuchi et al (2013)^[32]^	Japan	H	B	288	373	110	176	12	4
Smolen et al (2013)^[33]^	Europe, Latin America, Asia, etc	H	I	301	401	96	197	24	4
Gallo et al (2016)^[34]^	Italy	H	B	254	276	168	218	20	3

### 3.2 Network meta-analysis

#### 3.2.1 Network plot of 8 different fusion proteins combinations

**Figure 2:**
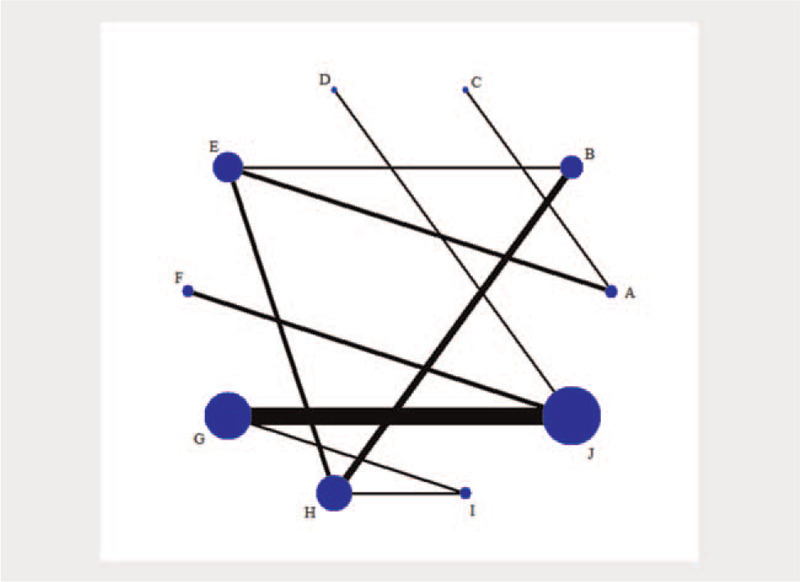
Network plot of different interventions for the treatment of RA. The size of the point in the network graph is proportional to the number of subjects, while the thickness of the line is proportional to the number of studies. A = placebo, B = MTX, C = ANA, D = ABA, E = ETN, F = ANA + MTX, G = ABA + MTX, H = ETN + MTX, I = ETN + placebo, J = MTX + placebo. ABA = abatacept, ANA = anakinra, ETN = etanercept, MTX = methotrexate.

Of the 19 publications involving 6812 patients, studies on the combination of placebo + MTX were the most frequent, while those on ANA and ABA alone were the least frequent. The placebo + MTX group had the highest number of subjects, while ABA had the lowest number of subjects (Fig. 2).

#### 3.2.2 Evidence contribution plot

**Figure 3:**
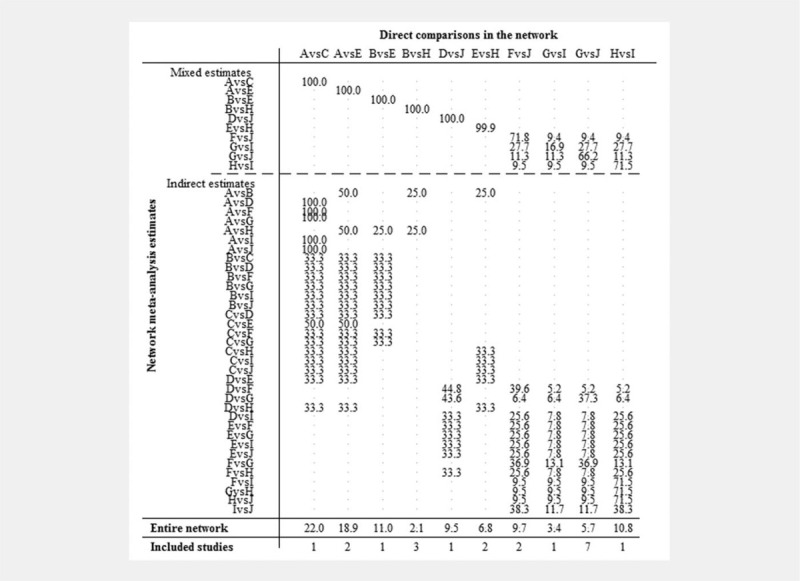
Evidence contribution plot. The matrix showed the effect of comparing the results of different control measures directly with the results of their network meta-analysis. A = placebo, B = MTX, C = ANA, D = ABA, E = ETN, F = ANA + MTX, G = ABA + MTX, H = ETN + MTX, I = ETN + placebo, J = MTX + placebo. ABA = abatacept, ANA = anakinra, ETN = etanercept, MTX = methotrexate.

The direct comparison of ETN alone and the combination of ETN + MTX had a 99% effect on the combined result. The direct comparison between ETN alone and ETN + MTX had a 25% effect on the indirect comparison between ANA and ABA. The direct comparison of ETN alone and ETN + MTX had a 6.8% effect on the results of the meta-analysis (Fig. 3).

#### 3.2.3 Predictive interval plot

**Figure 4:**
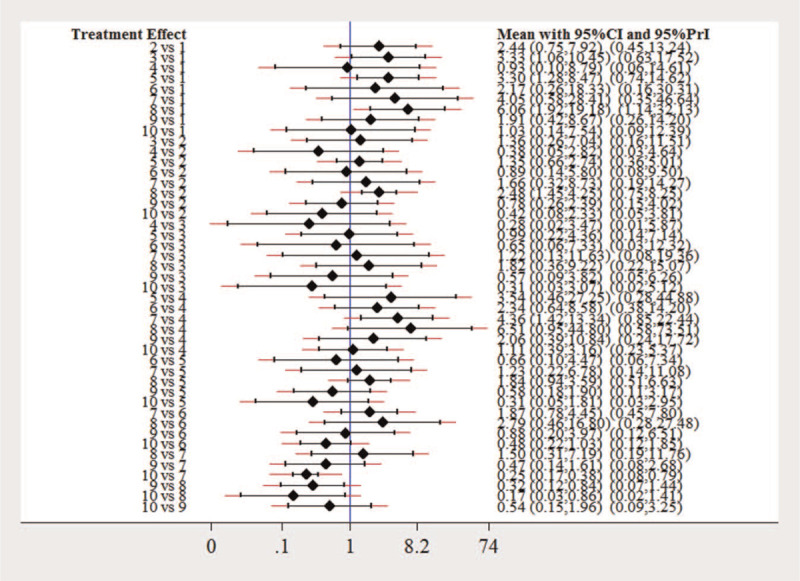
Network estimates of mean *OR*, their 95% CIs and prediction intervals (red extensions). A = placebo, B = MTX, C = ANA, D = ABA, E = ETN, F = ANA + MTX, G = ABA + MTX, H = ETN + MTX, I = ETN + placebo, J = MTX + placebo. ABA = abatacept, ANA = anakinra, ETN = etanercept, MTX = methotrexate.

It is showed that the pooled *OR* and 95% CI of RA improvement compared with placebo alone were 2.44(0.75, 7.92) for MTX, 3.33(1.06, 10.45) for ANA, 0.93(0.10, 8.79) for ABA, 3.30(1.28, 8.47) for ETN, 2.17(0.28, 18.33) for ANA + MTX, 4.05(0.58, 28.41) for ABA + MTX, 6.06(1.92, 19.18) for ETN + MTX, 1.91(0.42, 8.67) for ETN + placebo, and 1.03(0.14, 7.54) for MTX + placebo, respectively. The OR for the network estimates along with 95% CI and *PrI* is presented in Figure 4.

#### 3.2.4 Publication bias

**Figure 5:**
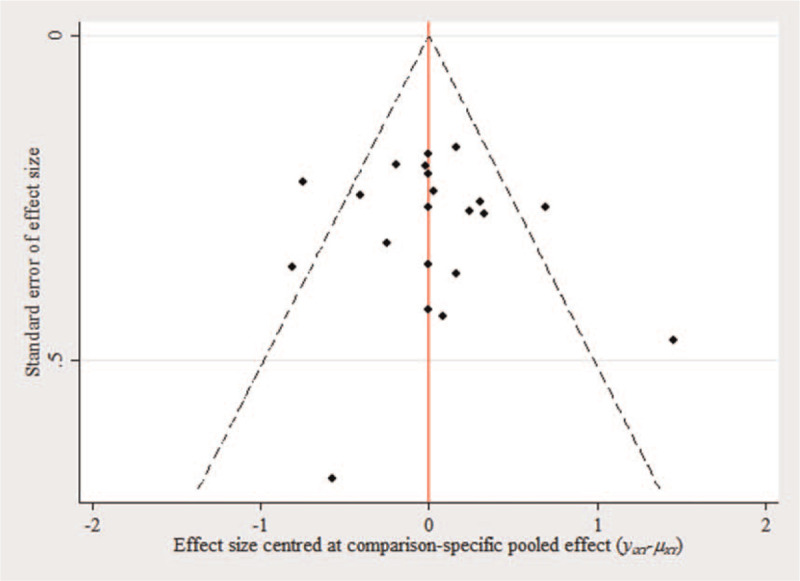
Funnel plot for publication bias in selected studies.

Regarding publication bias, all the outcomes in the study were not exactly symmetrical (Fig. 5), suggesting that the publication bias may have existed.

#### 3.2.5 Ranking plot

**Figure 6:**
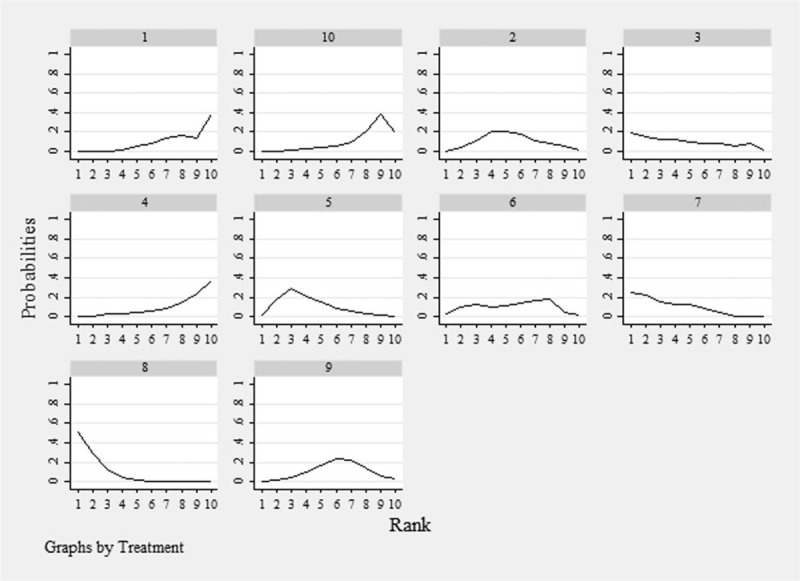
SUCRA for the cumulative probabilities. A = placebo, B = MTX, C = ANA, D = ABA, E = ETN, F = ANA + MTX, G = ABA + MTX, H = ETN + MTX, I = ETN + placebo, J = MTX + placebo. ABA = abatacept, ANA = anakinra, ETN = etanercept, MTX = methotrexate, SUCRA = surface values under the cumulative ranking.

The distribution of probabilities for each treatment being ranked for their efficacy in RA according to SUCRA values is presented in Table 2 and Figure 6. The order of SUCRA values for different aspirin combinations was as follows: ETN + MTX (91.6), ABA + MTX (76.6), ETN (67.1), ANA (64.9), MTX (51.8), ANA + MTX (50.1), ETN + placebo (42.2), MTX + placebo (19.1), ABA (18.5), and placebo (18.2). Therefore, the combination of ETN with MTX had the highest probability of being the best intervention option in terms of clinical efficacy.

**Table 2:** SUCRA of acute cerebral infarction treatments.

Treatment	SUCRA	Pr best	Mean
A	18.5	0.0	8.3
B	51.8	0.0	5.3
C	64.9	19.5	4.2
D	18.2	0.1	8.4
E	67.1	1.4	4.0
F	50.1	2.8	5.5
G	76.6	24.5	3.1
H	91.6	51.6	1.8
I	42.2	0.1	6.2
J	19.1	0.0	8.3

## 4 Discussion

This study analyzed 10 different combinations, which consisting of 3 fusion proteins or placebo or MTX. The resulting network shows that the combination of ETN with MTX had the highest probability of being the best treatment option of 8 combinations, and the SUCRA value was 91.6. ENT, ENT + MTX, and ANA were effective in the treatment of RA compared to placebo.

RA is 1 of the chronic inflammatory autoimmune diseases, leading to the function of other organs and tissues are damaged including symmetrical joints. Although the pathogenesis of RA still obscures, Matsuno et al^[35]^ has provided that tumor necrosis factor (TNF) is a key molecule that controls the inflammatory changes that occur in the RA synovium. In recent years, people have found biologics have been essential for increasing the likelihood of disease remission and low disease activity.^[36]^ Fusion protein is 1 of the biological agents, product a novel single protein with 2 partial functional properties, produced by genetic fusion of 2 or more genes.^[37]^ One part of the structure of the fusion protein provides molecular binding while the other part has certain functions.^[38]^ The functions part is called fc, which binding to specific receptors to achieve pharmaceutic properties of the construct.^[39]^ ETN is 1 of the fusion proteins that was first applied to the clinical treatment of RA.^[40]^ The pathogenesis of RA was classically viewed as the deregulation of Th1/Th2 balance, and that these cells secrete pro-inflammatory cytokines with a subset of TNF-pcr T helper cells.^[41]^ Recently, we have known that CD4 + CD25 high regulatory T cells (Treg cells) and newly reported IL-17-producing Th17 cells may challenge this classical theory. Therefore, the balance between Th17 and Treg, as well as Th1/Th2, has long been considered as 1 of the important factors in the treatment of autoimmune diseases including RA.^[42]^ Anti-TNF therapy may lead to the failure of the Treg suppression effect on cell proliferation to reduce Foxp3 mRNA expression.^[43]^ ETN, as a kind of TNF-α inhibitors, is the first TNF-α inhibitor for the treatment of RA. ETN can affect the production of various cytokines, chemokines, and proteases, possibly downstream of a pot of TNF-α cascade and regulating inflammatory processes in RA.^[44]^ Lina et al^[42]^ found that the combination of ETN and MTX can ameliorate the activity of the disease by regulating the differentiation or function of Th17 and Treg, and the patients treated with MTX alone showed no significant reversal of Th1/Th2 or Th17/Treg imbalances and no satisfactory remission after 12 weeks of treatment. Patients taking the combination of ETN and MTX reduced plasma interleukin-1 (IL-1) exposure, TNF-α, IL-6, IL-17, IL-23, and increased TGF-β. ANA is an IL-1 receptor antagonist (IL-1ra), which blocks IL-1, involved in inflammation; a protein associated with joint destruction for RA.^[45]^ ABA was composited of the extracellular domain and Fc portion of cytotoxic T lymphocyte-associated protein 4 (CTLA-4), which works by binding to the differential (CD)80/86 cluster on antigen-presenting cells (APC) and blocking the B7:CD28 interaction, then inhibiting joint inflammation.^[46]^

In this study, the combination of MTX and ETN agents had the highest probability of optimal treatment compared to other drugs. Iannone et al^[47]^ reached a similar conclusion that the combination of MTX and biologic agents more effective than MTX alone, which consistent with this study. The combination of ETN with MTX had the highest probability of being the best intervention option in terms of clinical efficacy. ENT, ENT + MTX, and ANA were effective in the treatment of RA compared to placebo. However, the adverse effects of biological agents have always been the focus of attention, and how to customize individualized biological therapies has always been valued by people. The combined use of drugs needs to consider the tolerance of RA patients. Inconsistencies may have an impact on the arguments raised by the use of only ACR20 criteria to evaluate the efficacy and quality of the original publication used. The results may be affected by inconsistent literature quality, the heterogeneity inherent in RA, and different ethnicities and sample sizes. Future research involving high-quality RCTs and large sample sizes is needed.

## 5 Conclusions

The combination of ETN and MTX had the highest probability of optimal treatment compared to other drugs; ENT, ENT + MTX, and ANA were effective in the treatment of RA compared to placebo.

## Acknowledgments

We gratefully acknowledge the assistance of our colleagues during the writing of this paper and the helpful advice of Fangyuan Yuand Liang Xu.

## Author contributions

**Conceptualization:** Mingcai Wu.

**Data curation:** Quanhai Wang, Hui Yuan.

**Formal analysis:** Quanhai Wang.

**Methodology:** Xiaohua Lu.

**Project administration:** Mingcai Wu, Mengjun Tao.

**Software:** Mingcai Wu, Mengjun Tao.

**Supervision:** Hui Yuan.

**Writing – original draft:** Mingcai Wu, Mengjun Tao.

**Writing – review & editing:** Mingcai Wu, Mengjun Tao.
